# P-1426. Comorbidities and Healthcare Resource Utilization Among People With HIV: Real-World Data From a Large Israeli Healthcare Provider

**DOI:** 10.1093/ofid/ofae631.1601

**Published:** 2025-01-29

**Authors:** Clara Weil, Sivan Gazit, Arlene Nugent, Liza Bronner Murrison, Moshe Hoshen, Ana Gabriela Pires dos Santos, Itzchak Levy

**Affiliations:** Kahn Sagol Maccabi Research and Innovation Center, Tel Aviv, Tel Aviv, Israel; Kahn Sagol Maccabi Research and Innovation Center, Tel Aviv, Tel Aviv, Israel; AbbVie, Etobicoke, Ontario, Canada; AbbVie, Etobicoke, Ontario, Canada; Kahn Sagol Maccabi Research and Innovation Center, Tel Aviv, Tel Aviv, Israel; AbbVie, Etobicoke, Ontario, Canada; Chaim Sheba Medical Center, Ramat Gan, Tel Aviv, Israel

## Abstract

**Background:**

Despite recent advances in HIV antiretroviral treatment (ART), unmet needs for people with HIV (PWH) remain. The study compared comorbid disease burden and healthcare resource utilization (HRU) among PWH vs those without HIV diagnosis, as well as by ART patterns.

**Methods:**

A retrospective cross-sectional study used anonymized electronic healthcare data from Israel’s Maccabi Healthcare Services (MHS; >2.7M members) to compare people living with HIV on 31/12/2022 with age-sex-matched controls without HIV diagnosis (1998−2022). PWH had to have ≥1 HIV diagnosis code(s) (1998−2022) and ≥1 dispensed ART prescription (2008−2022). Members enrolled for < 12 months were excluded. Sociodemographic characteristics, lifetime prevalence of comorbidities (exception: anxiety/depression, diagnosed and/or treated in 2022), and HRU (2022) were described. Among PWH dispensed ≥1 ART in 2022, patterns of 12-month percentage of days covered (PDC), discontinuation (≥90-day treatment gap), and interruption (resumed treatment after 6−89-day gap) were described.

**Results:**

Among 1973 PWH and 9865 controls, mean±SD age was 48.2±11.2 and 48.7±11.2 years, respectively, and 74.4% were male. A lower proportion of PWH (39.6%) vs controls (48.0%) had high residential socioeconomic status. Higher prevalence of comorbidities among PWH vs controls included anxiety/depression (26.2% vs 13.5%), liver disease (20.9% vs 12.5%), chronic kidney disease (14.9% vs 7.3%), cancer (7.2% vs 5.3%), hepatitis C virus (5.6% vs 0.1%), and hepatitis B virus (HBV; 3.4% vs 0.4%). PWH (vs controls) had a median of 10 primary-care visits/year (vs 7) and more frequent hospital admissions (≥1: 11.4% vs 6.6%) and emergency room visits (≥1: 26.5% vs 19.0%). Among PWH treated in 2022 (n=1907), 78.1% had PDC ≥90%. Discontinuation and interruption occurred among 7.2% and 69.1% of treated PWH, respectively. PWH with PDC < 90% (vs ≥90%) had younger age, lower socioeconomic status, higher prevalence of HBV diagnosis, and less frequent physician visits in prior 12 months.

**Conclusion:**

Despite high ART coverage, PWH in Israel experience an excess burden of comorbidities and HRU compared to age-sex-matched controls without HIV. Further research is needed to address the excess disease burden and better define treatment/care gaps among PWH.

**Disclosures:**

**Sivan Gazit, MD**, AbbVie: The relationship is not personal, AbbVie funded the current study, funding to the research institution where I work **Arlene Nugent, MSc**, Abbvie: Stocks/Bonds (Public Company) **Liza Bronner Murrison, PhD**, AbbVie: Stocks/Bonds (Public Company) **Ana Gabriela Pires dos Santos, PhD**, ABBVIE: Stocks/Bonds (Public Company)

